# Genome-wide profiling of angiogenic *cis*-regulatory elements unravels *cis*-regulatory SNPs for vascular abnormality

**DOI:** 10.1038/s41597-024-03272-6

**Published:** 2024-05-08

**Authors:** Lihui Jin, Zhenyuan Han, Xiaotong Mao, Jieru Lu, Bingqian Yan, Yiwen Lu, Lili Liang, Lin Wang, Yu Yu, Kun Sun

**Affiliations:** 1 Department of Pediatric Cardiology, Xinhua Hospital, School of Medicine, Shanghai Jiao Tong University, Shanghai, 200092 China; 2 Department of Oral and Maxillofacial Surgery, Peking University School and Hospital of Stomatology, Beijing, 100081 China; 3 Department of Gastroenterology, Changhai Hospital, Naval Medical University, Shanghai, 200433 China; 4 Department of Pediatrics, Lishui People’s Hospital, Lishui, 323050 China; 5 Department of NICU, Sir Run Run Shaw Hospital, School of Medicine, Zhejiang University, Hangzhou, 310016 China; 6 Department of Laboratory Medicine, Xinhua Hospital, School of Medicine, Shanghai Jiao Tong University, Shanghai, 200092 China; 7 Department of Pediatric Endocrinology/Genetics, Xinhua Hospital, School of Medicine, Shanghai Jiao Tong University, Shanghai, 200092 China; 8 Institute for Developmental and Regenerative Cardiovascular Medicine, Xinhua Hospital, School of Medicine, Shanghai Jiao Tong University, Shanghai, 200092 China

**Keywords:** Angiogenesis, Data mining, Development

## Abstract

Angiogenesis is extensively involved in embryonic development and requires complex regulation networks, whose defects can cause a variety of vascular abnormalities. *Cis*-regulatory elements control gene expression at all developmental stages, but they have not been studied or profiled in angiogenesis yet. In this study, we exploited public DNase-seq and RNA-seq datasets from a VEGFA-stimulated *in vitro* angiogenic model, and carried out an integrated analysis of the transcriptome and chromatin accessibility across the entire process. Totally, we generated a bank of 47,125 angiogenic *cis*-regulatory elements with promoter (marker by H3K4me3) and/or enhancer (marker by H3K27ac) activities. Motif enrichment analysis revealed that these angiogenic *cis*-regulatory elements interacted preferentially with ETS family TFs. With this tool, we performed an association study using our WES data of TAPVC and identified rs199530718 as a *cis*-regulatory SNP associated with disease risk. Altogether, this study generated a genome-wide bank of angiogenic *cis*-regulatory elements and illustrated its utility in identifying novel *cis*-regulatory SNPs for TAPVC, expanding new horizons of angiogenesis as well as vascular abnormality genetics.

## Introduction

Angiogenesis refers to the physiology process of forming new blood vessels from existing vascular networks, which is essential for vascular morphogenesis in almost all tissues in the body^1^. Any defects in angiogenesis can lead to vascular abnormality, including arteriovenous malformation^2^, congenital heart disease^3^ and infantile hemangioma^4^. Such kind of disease is common in children and adults, and may cause damage to health to varying degrees. Abnormal angiogenesis is also the hallmark of cancer and inflammatory and ischemic diseases^5^. It not only contributes to disease progression but serves as a promising target for drug treatment. Therefore, owing to its crucial role in human health, angiogenesis has gained substantial interest among researchers over the past decade.

With continued efforts and research, tremendous advances have been made in inspecting the complex molecular and genetic mechanisms underlying angiogenesis in human^6,7^. Particularly, endothelial cell proliferation, adhesion, migration and tube formation are thought to be key events for the angiogenic process^7^. Such endothelial cell behaviors can be regulated by external signaling components (e.g., *BMP6*^8^ and *DLL4*^9^), transcription factors (TFs) (e.g., *ETS1*^10^ and *FOXC1*^11^) and epigenetic enzymes (e.g., *EP300*^12^ and *EZH2*^13^), thereinto vascular endothelial growth factor A (VEGFA) exemplifies one of the most powerful regulators. As an extracellular signaling factor, VEGFA activates a cascade of endothelial cell gene transcription and controls almost every stage of angiogenesis in human physiology and diseases^14^.

In recent years, *cis*-regulation has emerged as an important mechanism of controlling gene expression in embryonic development, which requires the combinatorial interplay of TFs with defined *cis*-regulatory elements in the genome^15,16^. There is no unified definition of *cis*-regulatory elements yet, but in most cases they comprise promoters, enhancers, silencers and insulators^17^. Notably, comprehensive chromatin and epigenetic landscapes act as efficient tools for genome-wide characterization of *cis*-regulatory elements^18,19^. It is extremely noteworthy that *cis*-regulatory elements vary among different cell-types and tissues^20^, implying the necessity of compendium analysis of such elements during human embryo morphogenesis. Up till now, *cis*-regulatory elements have been carefully mapped in several human organs across early developmental time points, including the brain^21^, heart^22^ and adipose^23^. But few studies have concentrated on the field of angiogenesis, so that *cis*-regulatory elements associated with this process (hereafter referred to as ‘angiogenic *cis*-regulatory elements’) still remain obscure.

**Fig. 1:**
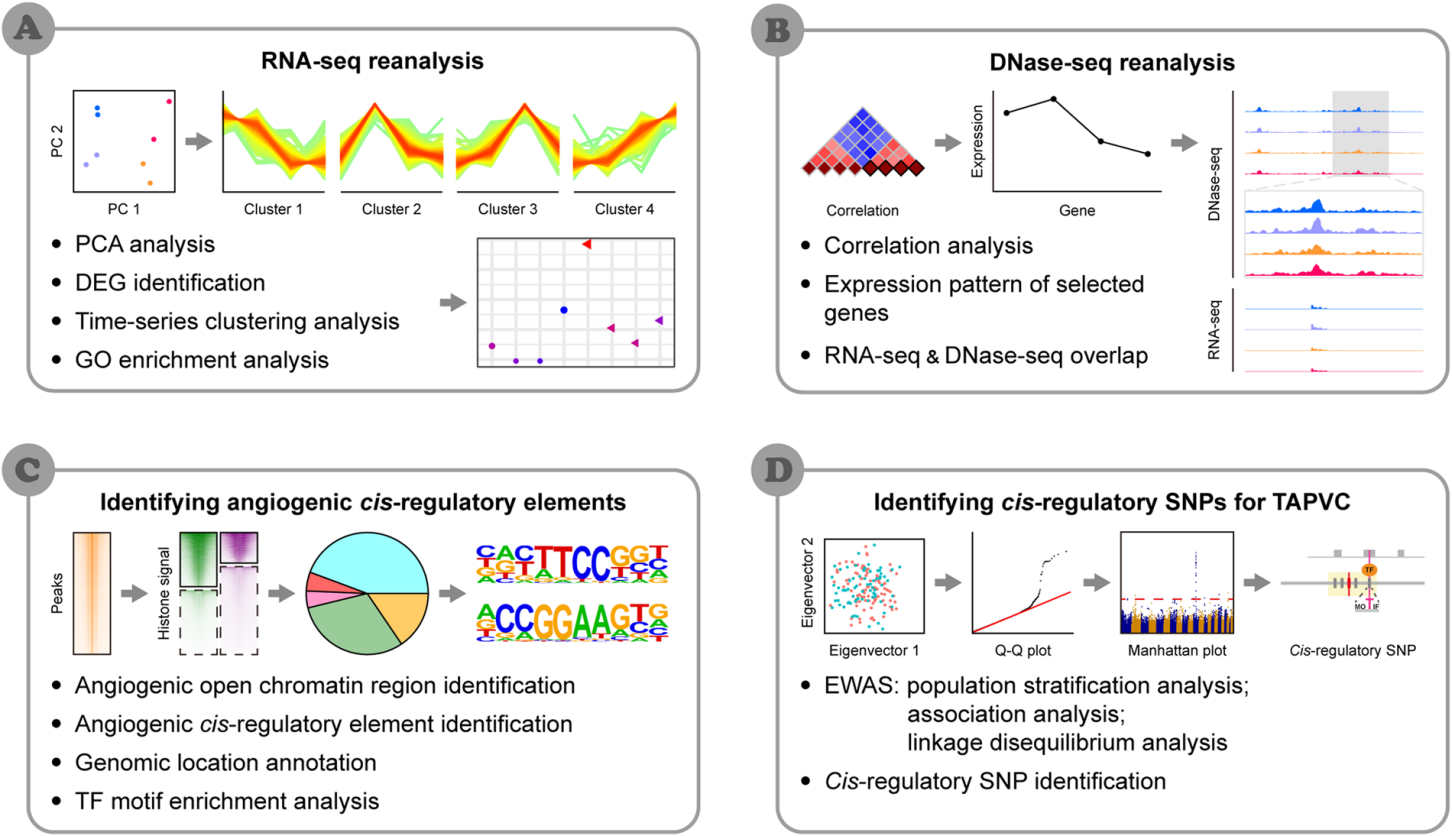
Overall workflow of this study.

In this study, to profile angiogenic *cis*-regulatory elements, we conducted an integrated analysis of the transcriptome and chromatin accessibility in a VEGFA-stimulated *in vitro* angiogenic model, as shown in Fig. 1. We generated a bank of 47,125 angiogenic *cis*-regulatory elements with promoter and/or enhancer activities. The angiogenic *cis*-regulatory elements were all located outside ‘gene desert’ regions and enriched for motifs of angiogenesis-relevant TFs. Using this bank, we performed a post exome-wide association study (EWAS) of total anomalous pulmonary venous connection (TAPVC) and found rs199530718 as a novel *cis*-regulatory single nucleotide polymorphism (SNP). These results provide a general landscape of *cis*-regulation in angiogenesis, and demonstrate the utility of angiogenic *cis*-regulatory elements in elucidating the genetics of vascular abnormality.

## Results

### Examining temporal transcriptome changes in VEGFA-stimulated HUVECs

**Fig. 2:**
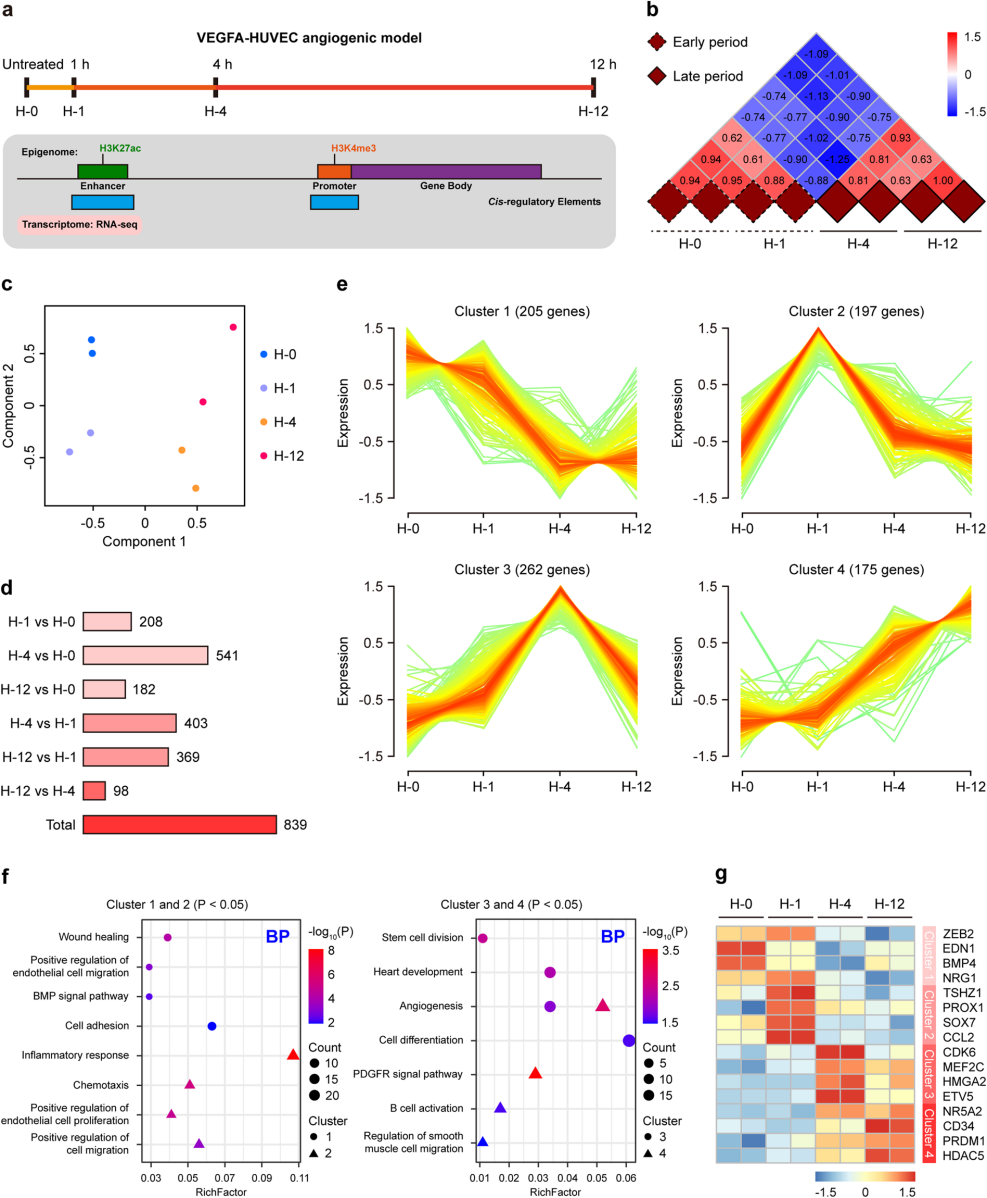
Temporal transcriptome dynamics of VEGFA-stimulated HUVECs. (**a**) Schematic illustration of the overall experimental design. Public genetic and epigenetic sequencing datasets of HUVECs before and after VEGFA stimulation for 1, 4 and 12 h were retrieved for analysis. (**b**) Correlation heat map of the DNase-seq tag densities in H-0, H-1, H-4 and H-12. Samples with similar chromatin accessibility were highlighted by solid or dashed borders. (**c**) PCA plot showing the first two components of H-0, H-1, H-4 and H-12. (**d**) Distribution of DEGs in pairwise comparison. (**e**) Time series patterns of DEGs during VEGFA stimulation. (**f**) Dot plots showing enriched GO BP terms of DEGs in Clusters 1 and 2 (left panel) as well as Clusters 3 and 4 (right panel), respectively. (**g**) Heat map of representative genes of Clusters 1 to 4.

The VEGFA-induced stimulation of human umbilical vein endothelial cells (HUVECs) (hereafter referred to as ‘VEGFA-HUVEC angiogenic model’) is an excellent *in vitro* system for studying the *cis*-regulation of angiogenesis^10,24–26^. To profile angiogenic *cis*-regulatory elements, we retrieved public genetic and epigenetic sequencing datasets of HUVECs before (H-0) and after VEGFA stimulation for 1 (H-1), 4 (H-4) and 12 (H-12) h (Fig. 2a)^10,24^. As open chromatin regions shared a similar genome-wide distribution with *cis*-regulatory elements^27^, we performed correlation analysis of the DNase-seq tag densities among these four stages to determine the relevance of their chromatin accessibility. Intriguingly, the entire stimulation process could be grouped into two time periods, that is, the early (H-0 and H-1) and late (H-4 and H-12) periods (Fig. 2b). This result, which had never been reported in VEGFA-HUVEC angiogenic model, demonstrated that the chromatin accessibility of HUVECs underwent temporal changes when stimulated by VEGFA. Given the landscapes of chromatin accessibility and gene expression were reciprocal causation in *in vitro* cardiogenesis^22^, we next examined the temporal transcriptome features of VEGFA-HUVEC angiogenic model. PCA analysis of the retrieved RNA-seq datasets discovered remarkable heterogeneity between the early and late periods of stimulation (Fig. 2c). Collectively, we speculated that in the late stimulation period, VEGFA might reprogrammed, or remodeled, HUVECs into a hitherto undescribed cell type.

We then screened differentially expressed genes (DEGs) in VEGFA-HUVEC angiogenic model to unveil the nature of H-4 and H-12. There were a total of 839 DEGs identified in the entire stimulation process (Fig. 2d), which was identical with Wang’s report^24^. These DEGs were further classified into four patterns (215 genes in Cluster 1, 197 in Cluster 2, 262 in Cluster 3, and 175 in Cluster 4) according to time-series clustering analysis (Fig. 2e, D**ata 1**). Genes in Clusters 1 and 2 showed high expression level in the early stimulation period, but were monotonically downregulated in the late period. By contrast, genes in Clusters 3 and 4 were continuously upregulated in the early stimulation period, and showed high expression level in the late period. We thus categorized the genes in Cluster 1 as H-0 specific enriched, Cluster 2 as H-1 specific enriched, Cluster 3 as H-4 specific enriched, and Cluster 4 as H-12 specific enriched, respectively.

Gene Ontology (GO) analysis of Cluster 1 or 2 gene set obtained GO Biological Process (BP) terms closely aligned with endothelial function (e.g., wound healing^28^, BMP signal pathway^29^ and inflammatory response^30^) (Fig. 2f-left panel). Further investigation of these two gene sets identified a DEG subset essential for endothelial identity, such as *ZEB2*^31^, *EDN1*^32^ and *PROX1*^33^ (Fig. 2g, Supplementary Fig. 1a). As for Cluster 3 or 4 gene set, we got GO BP terms related with progenitor cell function (e.g., angiogenesis^34^, stem cell division and cell differentiation) (Fig. 2f-right panel). Specially, these two gene sets were defined by progenitor cell markers like *CD34*^35^, *NR5A2*^36^ and *MEF2C*^37^ (Fig. 2g, Supplementary Fig. 1a). Taken together, our data suggested VEGFA reprogrammed HUVECs into a progenitor-like fate, and H-4 and H-12 exhibited angiogenic transcriptome features.

### Temporal transitions in VEGFA-stimulated HUVECs reflected by chromatin accessibility

**Fig. 3:**
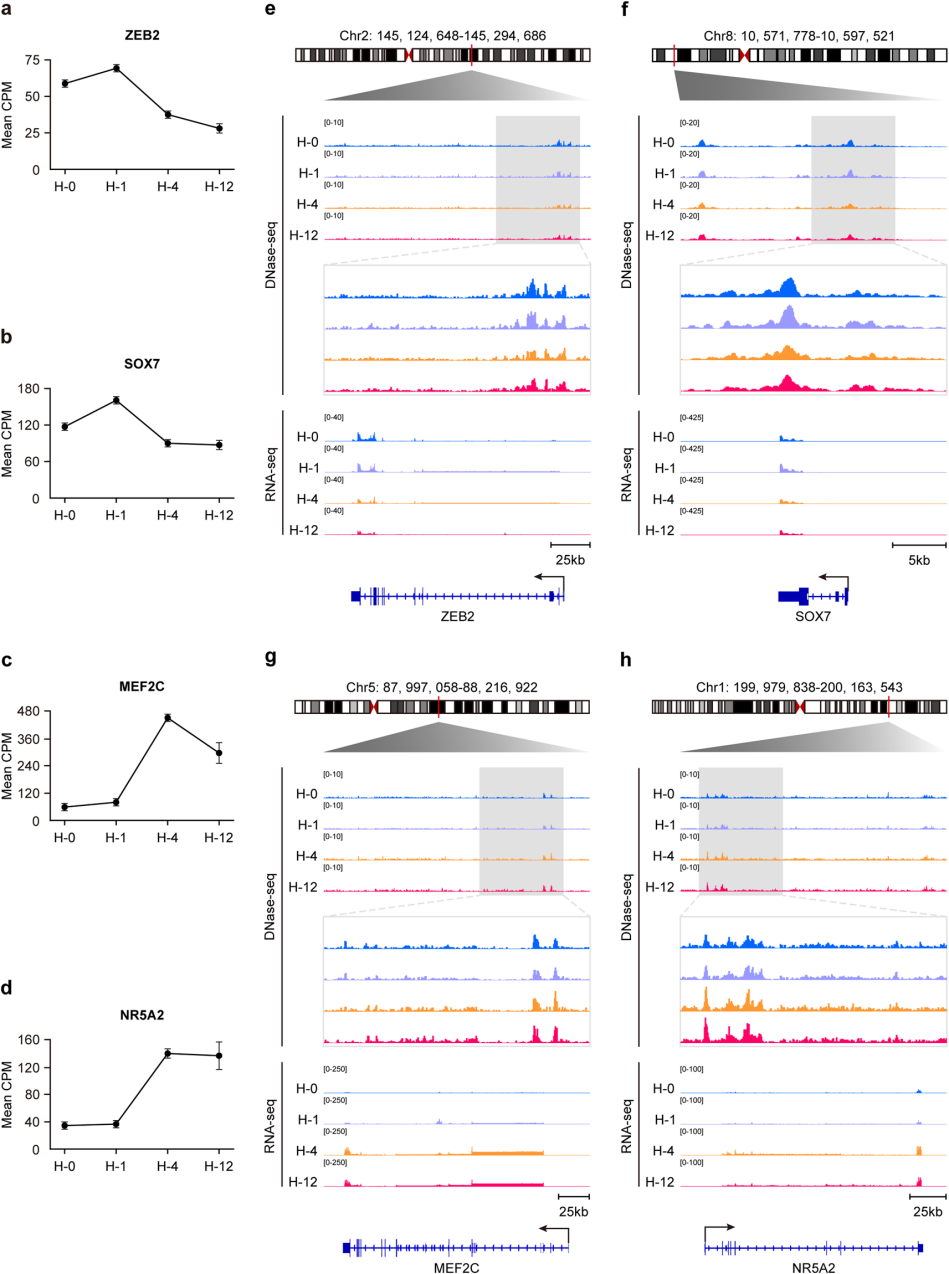
Temporal chromatin accessibility dynamics of VEGFA-stimulated HUVECs. (**a**–**d**) Normalized expression levels of *ZEB2* (**a**), *SOX7* (**b**), *MEF2C* (**c**) and *NR5A2* (**d**) during VEGFA stimulation, respectively. Data represented means ± SEM (n = 2 per group). **e-h** Normalized epigenetic and expression profiles at the *ZEB2* (**e**), *SOX7* (**f**), *MEF2C* (**g**) and *NR5A2* (**h**) loci during VEGFA stimulation, respectively.

Considering transcriptome as a readout of the *cis*-regulatory network, we investigated both the DNase-seq signals and the RNA-seq signals of DEGs at different stages in VEGFA-HUVEC angiogenic model. In Cluster 1 and 2 gene sets, we examined the *ZEB2* and *SOX7* gene loci due to their crucial roles in maintaining endothelial cell fate^31,38^. Compared with H-4 and H-12, H-0 and H-1 had higher enrichment of the DNase-seq signals at putative promoters and enhancers at both *ZEB2* and *SOX7* gene loci, which was consistent with the stages when these two genes were highly expressed (Fig. 3a,b,e,f). The *MEF2C* and *NR5A2* gene loci in Cluster 3 and 4 gene sets were then examined since they participated in pluripotency maintenance^36,37^. Their putative promoters and enhancers had more enriched DNase-seq signals in H-4 and H-12 than in H-0 or H-1, showing consistency with their respective mRNA expression dynamics (Fig. 3c,d,g,h). From these results, we observed temporal changes in chromatin accessibility related to VEGFA stimulation and correlated with gene transcriptions. The temporal transitions in VEGFA-stimulated HUVECs could be precisely reflected by the epigenetic dynamics. Specifically, the chromatin accessibility landscapes of H-4 and H-12 revealed the *cis*-regulatory network of angiogenesis.

### Identifying angiogenic *cis*-regulatory elements

**Fig. 4:**
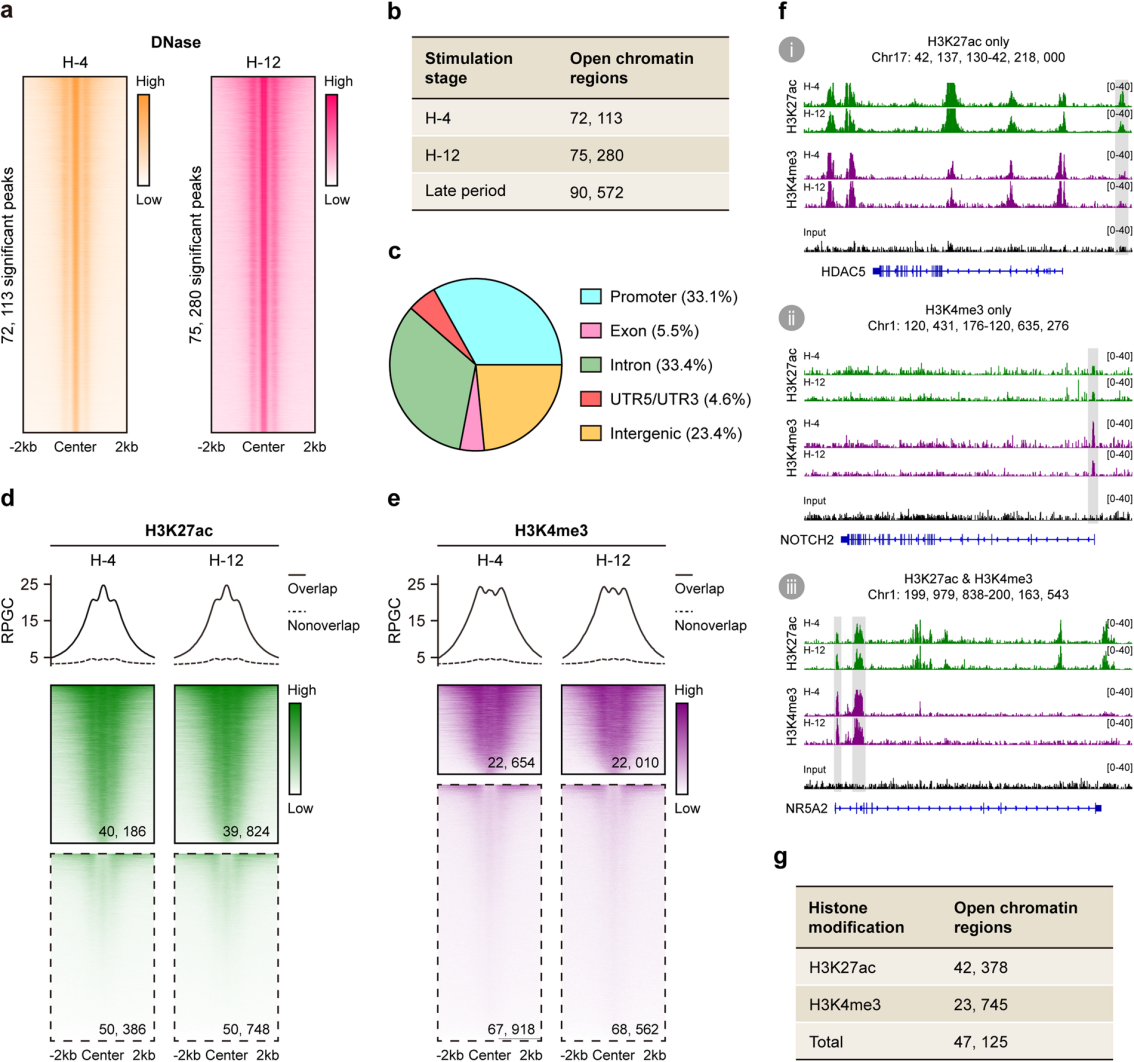
Epigenetic profiling predicted angiogenic *cis*-regulatory elements. (**a**) Read density heat maps showing normalized DNase-seq enrichments in H-4 (left panel) and H-12 (right panel) at their respective peak regions (center ± 2 kb). Peak regions in each heat map were represented as horizontal rows, and ranked by decreasing signal strength. (**b**) Number of open chromatin regions in H-4, H-12 and the whole late stimulation period. (**c**) Genomic distribution of angiogenic open chromatin regions. (**d**,**e**) Average profiles and read density heat maps showing normalized H3K27ac (**d**) and H3K4me3 (**e**) enrichments in H-4 and H-12 at angiogenic open chromatin regions (center ± 2 kb). Black solid and dashed borders were used to highlight genomic regions with and without ChIP-seq signals, respectively. (**f**) Typical examples of angiogenic open chromatin regions with histone modification of H3K27ac (i), H3K4me3 (ii) and both (iii). (**g**) Number of angiogenic open chromatin regions with H3K27ac and/or H3K4me3 modifications.

To identify angiogenic *cis*-regulatory elements, we searched for open chromatin regions in H-4 and H-12 based on the retrieved DNase-seq datasets. There were 72,113 significant DNase-seq peaks in H-4 as well as 75,280 in H-12, which were recognized as their respective open chromatin regions (Fig. 4a). Then a total of 90,572 angiogenic open chromatin regions was identified by merging the above genomic regions in H-4 and H-12 (Fig. 4b). Of all the angiogenic open chromatin regions, 29,929 (33.1%) were in promoters, 4,952 (5.5%) were in exons, 30,228 (33.4%) were in introns, 4,180 (4.6%) were in UTR5/UTR3, and 21,283 (23.4%) were in intergenic regions (Fig. 4c, D**ata 2**). The genomic distribution of angiogenic open chromatin regions was similar with that of other human tissues^39^, suggesting our strategy was accurate for profiling such genomic regions.

Since most of *cis*-regulatory elements in vertebrate genomes were enhancers and promoters^40^, we here confined angiogenic *cis*-regulatory elements to angiogenic open chromatin regions with enhancer or promoter activity. Public ChIP-seq datasets for two histone modifications, H3K27ac and H3K4me3, in H-4 and H-12 were retrieved in subsequent analysis. These two marks were widely used to label enhancers and promoters, respectively^41,42^. In H-4, there were 40,186 (44.4%) angiogenic open chromatin regions with H3K27ac enrichment and 22,654 (25.0%) with H3K4me3 enrichment (Fig. 4d,e). In H-12, there were 39,824 (44.0%) angiogenic open chromatin regions with H3K27ac enrichment and 22,010 (24.3%) with H3K4me3 enrichment (Fig. 4d,e). After merging these genomic regions in H-4 and H-12, we found 42,378 angiogenic open chromatin regions with enhancer activity (H3K27ac modification), and 23,745 with promoter activity (H3K4me3 modification) (Fig. 4g).

Noteworthy, most of the above open chromatin regions were monofunctional with either enhancer or promoter activity, as exemplified by the *HDAC5* and *NOTCH2* gene loci (Fig. 4fi,ii). The rest were bifunctional with both enhancer and promoter activities, as exemplified by the *NR5A2* gene locus (Fig. 4fiii). It was in accordance with a previous conclusion that some genomic regions might switch between enhancer and promoter signatures^43,44^. Thereby, we merged all open chromatin regions with H3K27ac and/or H3K4me3 modifications in H-4 and H-12, and obtained a total of 47,125 angiogenic *cis*-regulatory elements (Fig. 4g).

### Depicting epigenetic signatures of angiogenic *cis*-regulatory elements

**Fig. 5:**
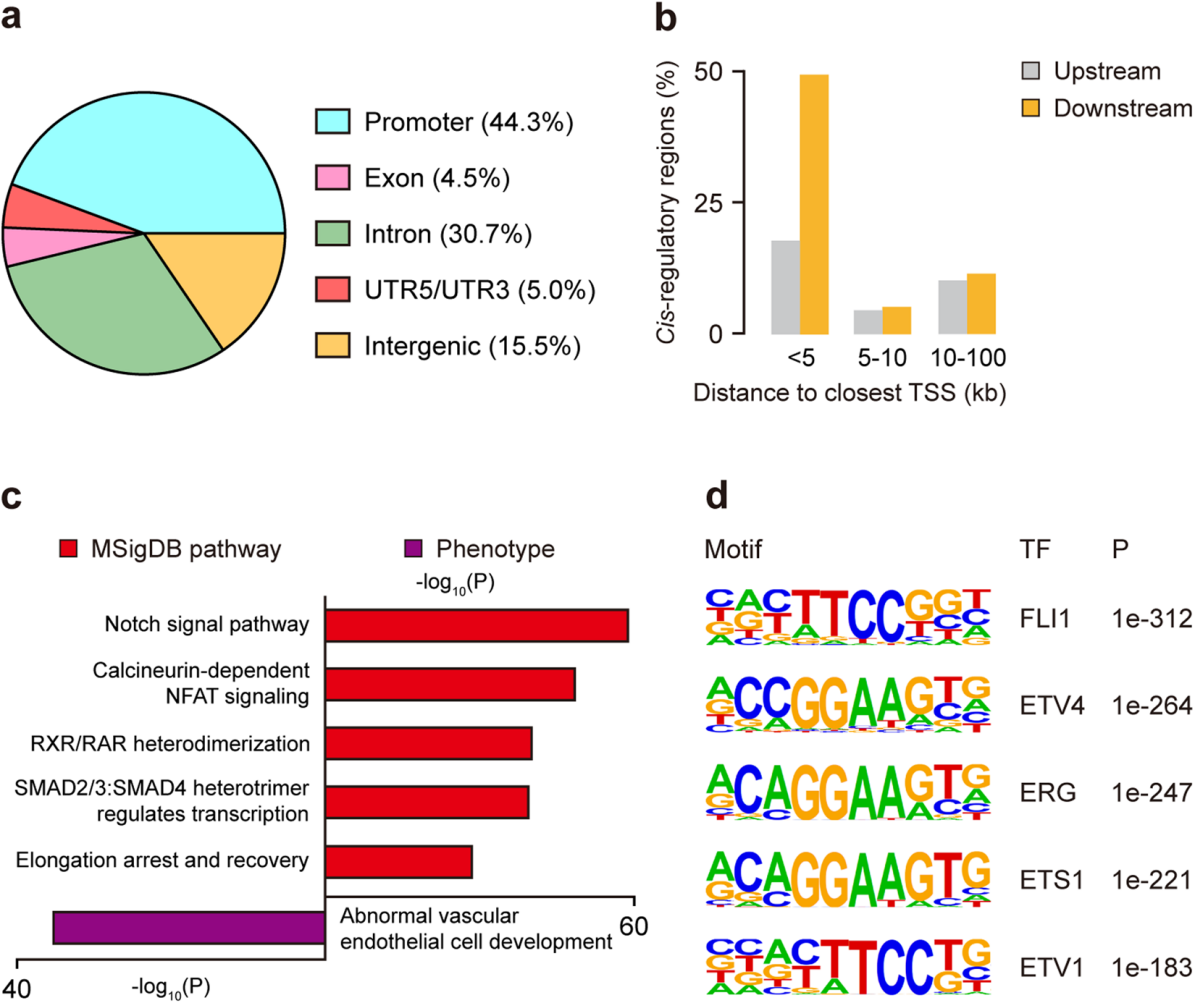
General features of angiogenic *cis*-regulatory elements. (**a**) Genomic distribution of angiogenic *cis*-regulatory elements. (**b**) Distance of each angiogenic *cis*-regulatory element from its nearest TSS. (**c**) Significant MSigDB pathways and phenotypes enriched in angiogenic *cis*-regulatory elements. (**d**) TF binding motifs enriched in angiogenic *cis*-regulatory elements.

We next compared angiogenic *cis*-regulatory elements with known features of the human genome. Of all the angiogenic *cis*-regulatory elements, 20,887 (44.3%) were in promoters, 2,111 (4.5%) were in exons, 14,458 (30.7%) were in introns, 2,361 (5.0%) were in UTR5/UTR3, and 7,308 (15.5%) were in intergenic regions (Fig. 5a, D**ata 3**). Obviously, the vast majority of angiogenic *cis*-regulatory elements were resided in genome noncoding regions, which conformed to the basic characteristic of regulatory DNA sequences^17^. On the other hand, we analyzed the genomic locations of angiogenic *cis*-regulatory elements according to gene annotation. 30,785 (65.3%) angiogenic *cis*-regulatory elements were found to locate within 5 kb upstream or downstream from their respective nearest TSSs, whereas the rest were distal from their respective neighboring genes (5 kb to 100 kb) (Fig. 5b). None of angiogenic *cis*-regulatory elements were settled in ‘gene desert’ regions (>500 kb that were devoid of protein coding genes). Collectively, our identified angiogenic *cis*-regulatory elements were almost noncoding sequences and might regulate gene transcription in angiogenesis *via* long-range interactions.

Further functional annotation of angiogenic *cis*-regulatory elements was conducted *via* GREAT. As expected, we found that the enriched MSigDB pathways were tightly related with angiogenesis, including Notch signal pathway^45^, elongation arrest and recovery^46^, NFAT signal pathway^47^ and transcription regulated by SMAD2/3:SMAD4 heterotrimer (Fig. 5c). These angiogenic *cis*-regulatory elements were also involved in abnormal vascular endothelial cell development (Fig. 5c), suggesting their important role in angiogenesis regulation. We then used HOMER to predict TFs that could potentially bind with angiogenic *cis*-regulatory elements. The TF with known motifs enriched in angiogenic *cis*-regulatory elements as well as high expression level in both H-4 and H-12 was recognized as a candidate. Over 15% of angiogenic *cis*-regulatory elements were enriched for motifs of TFs crucial for angiogenesis, such as *FLI1*^48^ (17.1%), *ETV4*^49^ (17.6%), *ERG*^50^ (20.6%), *ETS1*^10^ (14.2%) and *ETV1*^51^ (18.7%) (Fig. 5d). These five TFs also had persistently detectable mRNA levels in HUVECs during VEGFA stimulation (Supplementary Fig. 1b–f). With these results, we concluded that our identified angiogenic *cis*-regulatory elements contained comprehensive information on angiogenesis *cis*-regulation.

### Using angiogenic *cis*-regulatory elements as instrument for identifying *cis*-regulatory SNPs associated with TAPVC risk

**Fig. 6:**
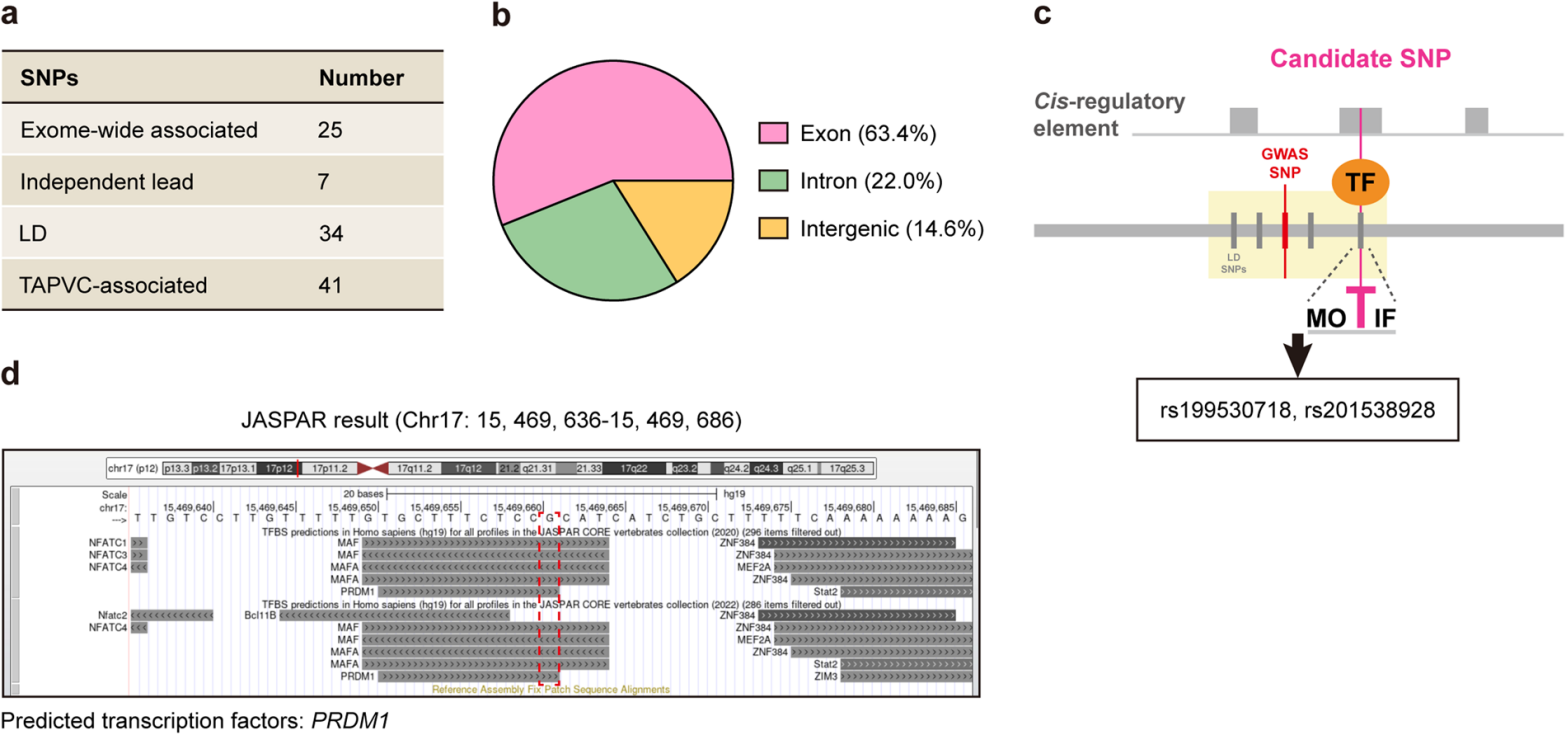
Using angiogenic *cis*-regulatory elements to find new *cis*-regulatory SNPs for TAPVC. (**a**) Number of exome-wide associated, independent lead, LD and TAPVC-associated SNPs. (**b**) Genomic distribution of TAPVC-associated SNPs. (**c**) Schematic illustration of the analysis pipeline. TAPVC-associated SNPs located in angiogenic *cis*-regulatory elements were recognized as *cis*-regulatory SNP candidates for TAPVC. (**d**) Predicted TFs bound to the rs199530718 locus *via* JASPAR database.

A relevant usage of angiogenic *cis*-regulatory elements was to guide post-EWAS studies by identifying vascular abnormality-associated *cis*-regulatory SNPs. We employed this instrument to screen *cis*-regulatory SNPs associated with TAPVC risk, a congenital heart disease mainly caused by aberrant angiogenesis^3^. The analysis pipeline had been put forward in our previous study^22^, and was shown in Supplementary Fig. 2a. Whole-exome sequencing (WES) data of 78 TAPVC cases and 100 controls passed quality control, and a subset of 121,107 common SNPs with high quality was selected for exome-wide association analysis. Of note, there was no population stratification between cases and controls (Supplementary Fig. 2b,c). We thus examined the exome-wide association in an additive logistic regression model without adjustment for any covariates. 25 SNPs showed statistical evidence of exome-wide association with TAPVC and were listed in **Data 4** (Fig. 6a, Supplementary Fig. 2d). To avoid any potential impact of linkage disequilibrium (LD) on the findings, we further set the threshold of *r*^*2*^ < 0.6 and obtained 7 independent lead SNPs among the exome-wide associated SNPs (Fig. 6a). LD expansion with a cutoff *r*^2^ value of 0.2 revealed another 34 SNPs that were in LD with at least one of the independent lead SNPs. Together, our EWAS discovered a total of 41 SNPs in association with TAPVC risk (Fig. 6a, D**ata 5**). Of all the TAPVC-associated SNPs, 26 (63.4%) were in exons, 9 (22.0%) were in introns, and 6 (14.6%) were in intergenic regions (Fig. 6b).

Next, our goal was to find if there were any *cis*-regulatory SNPs for TAPVC among 41 TAPVC-associated SNPs. Two TAPVC-associated SNPs were located within angiogenic *cis*-regulatory elements and thereby were recognized as *cis*-regulatory SNP candidates, namely rs199530718 and rs201538928 (Fig. 6c). rs199530718 was predicted to interact with *PRDM1* to form the ‘SNP-TF’ circuit (Fig. 6d), whereas rs201538928 was not located at any TF motifs (data not shown). *PRDM1* was a well-studied regulator of embryonic stem cell pluripotency and could affect the process of endothelial cell differentiation^52,53^. Once its DNA-binding motif was disrupted by rs199530718, *PRDM1* would fail to bind to the angiogenic *cis*-regulatory element containing the rs199530718-A allele. It could disable the *PRDM1*-mediated pluripotent transcription network in endothelial cells, making them hard to adopt the progenitor-like fate in angiogenesis. In summary, our analyses indicated that rs199530718 was a *cis*-regulatory SNP linked with TAPVC, validating the angiogenic *cis*-regulatory elements as an important tool to investigate vascular abnormality genetics.

## Discussion

This study carried out a comprehensive assessment of the transcriptome and chromatin signatures in VEGFA-HUVEC angiogenic model, and generated a bank of 47,125 angiogenic *cis*-regulatory elements. We used this bank to analyze the TAPVC-associated SNPs and discovered a novel *cis*-regulatory SNP for TAPVC, namely rs199530718. The risk allele rs199530718-A was predicted to disrupt the *PRDM1*-binding site on an angiogenic *cis*-regulatory element, thereby causing aberrant angiogenesis. Generally, this study provided a valuable tool for epigenetic dissection of angiogenesis and genetics of vascular abnormality.

Although the VEGFA-HUVEC angiogenic model is not new, the novelty of this study lies in its pure computational approach, integrating DNase-seq, RNA-seq and ChIP-seq datasets deposited on the Gene Expression Omnibus (GEO). Previous analysis of these datasets, combined with cellular assay, has revealed VEGFA-induced transcriptional responses and VEGFA-responsive enhancers in endothelial cells. These results not only elucidated the basic features of VEGFA-HUVEC angiogenic model but also suggested that it was reliable for mimicking angiogenesis. Here, we focused on the angiogenic process rather than the endothelial cell itself. Specifically, based on VEGFA-HUVEC angiogenic model, our computational approach re-exploited the above GEO datasets to study the *cis*-regulation of angiogenesis.

*In vitro* differentiation models recapitulating organ and tissue development have been widely applied to explore the *cis*-regulation of embryonic morphogenesis. Typical examples include *in vitro* differentiation of cardiomyocytes^22,54^, retina^55^ and hypothalamic neurons^23^. In this study, VEGFA-HUVEC angiogenic model was used to investigate the *cis*-regulation of angiogenesis. Although this model had been established over a decade, we reached a previously unreported conclusion that HUVECs would adopt a progenitor-like fate in the late stimulation period. Concretely, H-1 showed the endothelial transcriptome characteristics, whereas H-4 and H-12 had enriched BP terms related to angiogenesis and multi-lineage differentiation potential. Under this condition, VEGFA might act as a chemical molecule to reprogram HUVECs into progenitor-like cells to trigger angiogenesis. It was a reasonable speculation as the reprogramming effect of VEGFA had already been observed on endothelial cells in hepatocellular carcinoma^56^. Besides, our conclusion conformed to the classic sprouting angiogenesis theory, which highlighted the indispensable role of endothelial progenitor cells during the angiogenic process^34^. Therefore in subsequent analysis, diverse epigenetic datasets of H-4 and H-12 were integratedly examined to profile angiogenic *cis*-regulatory elements.

It is worth mentioning that none of the techniques available today can provide direct information on genomic *cis*-regulatory elements. Thus in the study, we adopted an indirect profiling method by firstly mapping angiogenic open chromatin regions with enhancer and/or promoter activities. Briefly, the angiogenic open chromatin regions were detected *via* DNase-seq datasets, and the enhancer or promoter activity was detected *via* histone ChIP-seq datasets. This method is mature for profiling genomic *cis*-regulatory elements, of note, as evidenced by recent achievements in the field of somite and heart development^22,57^. On the other hand, our identified angiogenic *cis*-regulatory elements were enriched for DNA-binding motifs of ETS family TFs (e.g., *FLI1*, *ETV4*, *ERG* and *ETS1*). TFs belonging to the ETS family are master regulators of endothelial cell gene transcription and participate actively in angiogenic signal transduction^46^. Depleting these TFs can impair angiogenesis and lead to vascular abnormality during embryogenesis^58^. Moreover, Zhang *et al*.^10^ have demonstrated that almost all of the angiogenic enhancers contain ETS TF motif sequences. All these evidences prove that the angiogenic *cis*-regulatory element bank we generated is comprehensive and reflects the nature of angiogenesis.

The angiogenic *cis*-regulatory element bank serves as a valuable resource for investigating the angiogenesis and genetics of vascular abnormality. In this study, the bank was used in a post-EWAS analysis to annotate potential *cis*-regulatory functions of the TAPVC-associated SNPs. Typical examples of such application can be seen in the etiological study of common diseases, such as ventricular septal defect^22^, acute lymphoblastic leukemia^59^ and Parkinson’s disease^60^. Such kind of *cis*-regulatory element banks also contributes to researches including gene transcription control^61^, targeted gene finding^62^, multigenome DNA sequence conservation^63^ and gene therapy^64^. For instance, Lee *et al*. screened proximal *cis*-regulatory elements in the *IL-10* gene loci of Th1 and Th2 cells, and reported a new enhancer that can regulate *IL-10* expression in distinct T helpers. Here, while we advocate the use of the angiogenic *cis*-regulatory element bank to recognize *cis*-regulatory SNPs for vascular abnormality, we also emphasize that, the ‘SNP-TF’ circuit is a vital clue to prioritize that TF for future follow-up studies.

To sum up, our integrated genetic and epigenetic analysis has generated a genome-wide bank of angiogenic *cis*-regulatory elements. Browsing the bank enables recognition and understanding of novel *cis*-regulatory SNPs linked with TAPVC. This study is limited by the lack of evidence from molecular and cellular experiments, which hinders our efforts to further explore angiogenic *cis*-regulatory elements. Nevertheless, the angiogenic *cis*-regulatory element bank and the study itself have provided a tool for investigating the *cis*-regulation of angiogenesis, and contribute to understand genetics of vascular abnormality.

## Methods

### High-throughput datasets

For this study, high-throughput data from HUVECs before and/or after VEGFA stimulation were reanalyzed. Raw FASTQ files for RNA-seq and DNase-seq were downloaded from GEO series GSE41166^10,65^, and for H3K27ac and H3K4me3 ChIP-seq were downloaded from GEO series GSE109626^24,66^. Before alignment, raw sequencing reads were trimmed to generate clean reads *via* Trim Galore (version 0.6.7) with parameters ‘-q 20 --length 20 --stringency 4 --e 0.1’.

### Reanalysis of RNA-seq datasets

Clean reads were aligned to the hg19 reference genome *via* Hisat2 (version 2.2.1) with default parameters^67^, and then SAMtools (version 1.9) was used to remove duplicate reads^68^. Total reads that overlapped the exons of the genes were counted *via* HTSeq (version 0.13.5) with parameters ‘-s n -t exon’^69^. Raw gene expression values were computed as counts per million mapped reads (CPM).

For visualization, post-filtered BAM files were normalized and converted to BIGWIG format *via* deepTools2 bamCoverage with parameters ‘--normalizeUsing RPGC --effectiveGenomeSize 2864785220 --binSize 10’^70^. Gene tracks were visualized *via* Integrative Genomics Viewers (IGV).

For principal component analysis (PCA), principal components of gene expression data from all samples were calculated *via* R function ‘prcomp’. The first two components were then visualized *via* R package ‘ggplot2’.

For differential analysis, differential expression was assessed by performing all pairwise comparisons among samples. R package ‘DESeq 2’ was used to identify DEGs following the criteria of |log2 (fold change)| ≥ 0.58 and adjust *p* ≤ 0.01. Time-series clustering of DEGs was analyzed *via* R package ‘Mfuzz’ with parameter ‘c = 4’.

For functional annotation, GO enrichment analysis for each time-series cluster of DEGs was carried out *via* DAVID database (https://david.ncifcrf.gov/)^71^. The GO terms with *p* < 0.05 were considered as significant and visualized *via* R package ‘ggplot2’.

### Reanalysis of DNase-seq datasets

Clean reads were aligned to hg19 genome *via* Bowtie2 (version 2.4.4) with default parameters^72^. Aligned BAM files were then processed to remove low quality mapped and duplicate reads. Peak calling was performed *via* MACS2 (version 2.1.1.20160309) with parameters ‘--nomodel --shift 100 --extsize 200 -q 0.05’.

For visualization, the pipelines of generating BIGWIG files and visualizing gene tracks were the same as those for RNA-seq datasets. Particularly, significant DNase-seq peaks were visualized *via* deepTools2 plotHeatmap.

For correlation analysis, genome-wide correlation matrix was calculated *via* deepTools2 multiBamSummary and plotCorrelation with parameters ‘--corMethod pearson --binSize 10000’. Post-filtered BAM files of all samples were imported as inputs. The correlation heat map was generated *via* R package ‘pheatmap’.

### Reanalysis of ChIP-seq datasets

The analysis pipeline for ChIP-seq reads was the same as that for DNase-seq datasets. Particularly, broad peaks were called *via* MACS2 with parameters ‘—broad --broad-cutoff 0.1’ and then visualized *via* IGV.

For read density analysis, the read density matrix was counted *via* deepTools2 computeMatrix with parameters ‘--referencePoint center -a 2000 -b 2000’, and then was visualized *via* deepTools2 plotHeatmap.

### Identification and annotation of open chromatin regions

The DNase-seq peaks in each sample constituted the initial set of its respective open chromatin regions. Genomic location annotation of open chromatin regions was performed *via* R package ‘ChIPseeker’.

### Identification and annotation of *cis*-regulatory elements

The DNase-seq peaks that had an overlap with H3K27ac and/or H3K4me3 peaks in each sample constituted the initial set of its respective *cis*-regulatory elements. Genomic location annotation of *cis*-regulatory elements was performed *via* R package ‘ChIPseeker’. Pathway and other enriched functions were predicted *via* GREAT (version 3.0.0; http://great.stanford.edu/public/html/)^73^. The enriched terms with *p* < 0.05 were considered as significant. TF motif enrichment analysis was performed *via* HOMER with the algorithm ‘findMotifsGenome.pl’^74^. The enriched motifs with *p* < 1 × 10^−20^ were considered as significant.

### Exome-wide association analysis

WES data of 78 TAPVC cases and 100 healthy controls were derived from our previous study^3^. All of the study population was unrelated and recruited from Xinhua Hospital affiliated to Shanghai Jiao Tong University. Before enrollment, written informed consents were signed by participants or their guardians.

For individual quality control, no individuals were filtered out owing to sex discrepancies or low genotyping rate (<95%). For SNP quality control, SNPs were excluded if they were located on sex chromosomes, if their call rate was <95%, if the minor allele frequency (MAF) was <0.05 among controls, or if the *p* value in Hardy-Weinberg equilibrium test was < 1 × 10^−5^ among controls. A total of 121,107 high-quality SNPs passed quality control testing and was included for exome-wide association analysis.

For population stratification analysis, PCA of 78 TAPVC cases and 100 controls was performed *via* PLINK (version 1.90) using all high-quality SNPs^75^. The first two eigenvectors were visualized *via* R package ‘ggplot2’.

For association analysis, exome-wide associations were assessed in an additive logistic regression model *via* PLINK without adjustment for any covariates. SNPs with *p* < 1 × 10^−5^ were considered as exome-wide associated. The quantile-quantile (Q-Q) plot and the Manhattan plot were both generated *via* R package ‘qqman’.

For LD analysis, independent lead SNPs were extracted from exome-wide associated SNPs which were independent from each other at *r*^*2*^ < 0.6. LD SNPs were extracted from high-quality SNPs which were in LD (*r*^*2*^ > 0.2) with at least one independent lead SNP. TAPVC-associated SNPs were the union of independent lead SNPs and LD SNPs. Particularly, functional annotation of the TAPVC-associated SNPs was carried out *via* ANNOVAR^76^.

### Supplementary information

Supplementary Information

## Data Availability

All data analyzed in this study were summarized in Supplementary Table 1. GEO datasets were available at https://ncbi.nlm.nih.gov/geo/. All high-throughput tools and R packages used in this study were public resources as described in Methods. All data generated were accessible on the *figshare* repository (ref. ^77^), as listed below: 1. Data 1: DEGs identified in the VEGFA-HUVEC angiogenic model. 2. Data 2: angiogenic open chromatin regions. 3. Data 3: angiogenic cis-regulatory elements. 4. Data 4: exome-wide associated SNPs for TAPVC. 5. Data 5: TAPVC-associated SNPs.

## Code availability

No custom code was used.

## References

[1] Liu T, Zhang L, Joo D, Sun S-C (2017). NF-κB signaling in inflammation. Signal Transduct. Target. Ther..

[2] Lee H-W (2021). Role of venous endothelial cells in developmental and pathologic angiogenesis. Circulation.

[3] Shi X (2018). Next-generation sequencing identifies novel genes with rare variants in total anomalous pulmonary venous connection. EBioMedicine.

[4] Queisser A, Seront E, Boon LM, Vikkula M (2021). Genetic basis and therapies for vascular anomalies. Circ. Res..

[5] Potente M, Gerhardt H, Carmeliet P (2011). Basic and therapeutic aspects of angiogenesis. Cell.

[6] Rogers, M. S. & D’Amato, R. J. Common polymorphisms in angiogenesis. *Cold Spring Harb. Perspect. Med*. **2** (2012). 10.1101/cshperspect.a006510 PMC3543099 23125197

[7] Lamalice L, Le Boeuf F, Huot J (2007). Endothelial cell migration during angiogenesis. Circ. Res..

[8] Pulkkinen HH (2021). BMP6/TAZ-Hippo signaling modulates angiogenesis and endothelial cell response to VEGF. Angiogenesis.

[9] Pitulescu ME (2017). Dll4 and Notch signalling couples sprouting angiogenesis and artery formation. Nat. Cell Biol..

[10] Zhang B (2013). A dynamic H3K27ac signature identifies VEGFA-stimulated endothelial enhancers and requires EP300 activity. Genome Res..

[11] Prasitsak T (2015). Foxc1 is required for early stage telencephalic vascular development. Dev. Dyn..

[12] Yao TP (1998). Gene dosage-dependent embryonic development and proliferation defects in mice lacking the transcriptional integrator p300. Cell.

[13] Lu C (2010). Regulation of tumor angiogenesis by EZH2. Cancer Cell.

[14] Leung DW, Cachianes G, Kuang WJ, Goeddel DV, Ferrara N (1989). Vascular endothelial growth factor is a secreted angiogenic mitogen. Science.

[15] Arnone MI, Davidson EH (1997). The hardwiring of development: organization and function of genomic regulatory systems. Development.

[16] Ghazi A, VijayRaghavan KV (2000). Developmental biology. Control by combinatorial codes. Nature.

[17] Schmitz RJ, Grotewold E, Stam M (2022). Cis-regulatory sequences in plants: Their importance, discovery, and future challenges. Plant Cell.

[18] de Laat W, Duboule D (2013). Topology of mammalian developmental enhancers and their regulatory landscapes. Nature.

[19] Klemm SL, Shipony Z, Greenleaf WJ (2019). Chromatin accessibility and the regulatory epigenome. Nat. Rev. Genet..

[20] Nott A (2019). Brain cell type-specific enhancer-promoter interactome maps and disease-risk association. Science.

[21] Song M (2019). Mapping cis-regulatory chromatin contacts in neural cells links neurop sychiatric disorder risk variants to target genes. Nat. Genet..

[22] Jin L (2022). Integrated genomic analysis identifies novel low-frequency cis-regulatory variant rs2279658 associated with VSD risk in Chinese children. Front. Cell Dev. Biol..

[23] Joslin AC (2021). A functional genomics pipeline identifies pleiotropy and cross-tissue effects within obesity-associated GWAS loci. Nat. Commun..

[24] Wang S (2019). A dynamic and integrated epigenetic program at distal regions orchestrates transcriptional responses to VEGFA. Genome Res..

[25] Abhinand CS (2023). Temporal phosphoproteomic analysis of VEGF-A signaling in HUVECs: an insight into early signaling events associated with angiogenesis. J. Cell Commun. Signal..

[26] Sunitha P (2019). Temporal VEGFA responsive genes in HUVECs: Gene signatures and potential ligands/receptors fine-tuning angiogenesis. J. Cell Commun. Signal..

[27] Ackermann AM, Wang Z, Schug J, Naji A, Kaestner KH (2016). Integration of ATAC-seq and RNA-seq identifies human alpha cell and be ta cell signature genes. Mol. Metab..

[28] Malinda KM (1998). Thymosin alpha 1 stimulates endothelial cell migration, angiogenesis, and wound healing. J. Immunol..

[29] Han O, Pak B, Jin S-W (2021). The role of BMP signaling in endothelial heterogeneity. Front. Cell Dev. Biol..

[30] Ambrozova G (2016). Nitro-oleic acid inhibits vascular endothelial inflammatory responses and the endothelial-mesenchymal transition. Biochim. Biophys. Acta..

[31] Liu Y (2020). MicroRNA-200c-3p inhibits proliferation and migration of renal artery endothelial cells by directly targeting ZEB2. Exp. Cell Res..

[32] Schönbach, C. *et al*. NKX2-3 transcriptional regulation of endothelin-1 and VEGF signaling in human intestinal microvascular endothelial cells. *PLoS ONE* **6** (2011). 10.1371/journal.pone.0020454 PMC3102722 21637825

[33] Hong Y-K (2002). Prox1 is a master control gene in the program specifying lymphatic end othelial cell fate. Dev. Dyn..

[34] Laurenzana A (2015). Endothelial progenitor cells in sprouting angiogenesis: proteases pave the way. Curr. Mol. Med..

[35] Sidney LE, Branch MJ, Dunphy SE, Dua HS, Hopkinson A (2014). Concise review: evidence for CD34 as a common marker for diverse progenitors. Stem Cells.

[36] Festuccia N, Owens N, Chervova A, Dubois A, Navarro P (2021). The combined action of Esrrb and Nr5a2 is essential for murine naïve pluripotency. Development.

[37] Vitali C, Tripodo C, Colombo MP (2015). MEF2C and SOCS2 in stemness regulation. Oncoscience.

[38] Yao Y, Yao J, Boström KI (2019). SOX transcription factors in endothelial differentiation and endothelial-mesenchymal transitions. Front. Cardiovasc. Med..

[39] Li J (2018). Accurate annotation of accessible chromatin in mouse and human primordial germ cells. Cell Res..

[40] Lu F (2016). Establishing chromatin regulatory landscape during mouse preimplantation development. Cell.

[41] Creyghton MP (2010). Histone H3K27ac separates active from poised enhancers and predicts developmental state. Proc. Nat. Acad. Sci. USA.

[42] Lauberth SM (2013). H3K4me3 interactions with TAF3 regulate preinitiation complex assembly and selective gene activation. Cell.

[43] Leung D (2015). Integrative analysis of haplotype-resolved epigenomes across human tissues. Nature.

[44] Roadmap Epigenomics Consortium (2015). Integrative analysis of 111 reference human epigenomes. Nature.

[45] Ramasamy SK, Kusumbe AP, Wang L, Adams RH (2014). Endothelial notch activity promotes angiogenesis and osteogenesis in bone. Nature.

[46] Chen J (2017). VEGF amplifies transcription through ETS1 acetylation to enable angiogenesis. Nat. Commun..

[47] Graef IA, Chen F, Chen L, Kuo A, Crabtree GR (2001). Signals transduced by Ca(2+)/calcineurin and NFATc3/c4 pattern the developing vasculature. Cell.

[48] Toyama T (2017). The impact of transcription factor Fli1 deficiency on the regulation o f angiogenesis. Exp. Dermatol..

[49] Harel S (2021). ETS1, ELK1, and ETV4 transcription factors regulate angiopoietin-1 signaling and the angiogenic response in endothelial cells. Front. Physiol..

[50] Shah AV, Birdsey GM, Randi AM (2016). Regulation of endothelial homeostasis, vascular development and angiogenesis by the transcription factor ERG. Vascul. Pharmacol..

[51] Petit FG, Salas R, Tsai M-J, Tsai SY (2004). The regulation of COUP-TFII gene expression by Ets-1 is enhanced by the steroid receptor co-activators. Mech. Ageing Dev..

[52] Chu LF, Surani MA, Jaenisch R, Zwaka TP (2011). Blimp1 expression predicts embryonic stem cell development *in vitro*. Curr. Biol..

[53] Niimi K, Nakae J, Inagaki S, Furuyama T (2021). FOXO1 represses lymphatic valve formation and maintenance *via* PRDM1. Cell Rep..

[54] Bertero A (2019). Dynamics of genome reorganization during human cardiogenesis reveal an RBM20-dependent splicing factory. Nat. Commun..

[55] Xie H (2020). Chromatin accessibility analysis reveals regulatory dynamics of developing human retina and hiPSC-derived retinal organoids. Sci. Adv..

[56] Sharma A (2020). Onco-fetal reprogramming of endothelial cells drives immunosuppressive macrophages in hepatocellular carcinoma. Cell.

[57] Mok GF (2021). Characterising open chromatin in chick embryos identifies cis-regulatory elements important for paraxial mesoderm formation and axis extension. Nat. Commun..

[58] Wei G (2009). Ets1 and Ets2 are required for endothelial cell survival during embryonic angiogenesis. Blood.

[59] Yang H (2022). Noncoding genetic variation in GATA3 increases acute lymphoblastic leukemia risk through local and global changes in chromatin conformation. Nat. Genet..

[60] Lee AJ (2023). Characterization of altered molecular mechanisms in Parkinson’s disease through cell type-resolved multiomics analyses. Sci. Adv..

[61] Lee C-G (2009). A distal cis-regulatory element, CNS-9, controls NFAT1 and IRF4-mediated IL-10 gene activation in T helper cells. Mol. Immunol..

[62] Zhang W (2005). *Cis*-regulatory element based targeted gene finding: genome-wide identification of abscisic acid- and abiotic stress-responsive genes in Arabidopsis thaliana. Bioinformatics.

[63] Kuntz SG (2008). Multigenome DNA sequence conservation identifies Hox *cis*-regulatory elements. Genome Res..

[64] Antoniou P (2022). Base-editing-mediated dissection of a γ-globin *cis*-regulatory element for the therapeutic reactivation of fetal hemoglobin expression. Nat. Commun..

[65] (2013). NCBI Sequence Read Archive.

[66] (2019). NCBI Sequence Read Archive.

[67] Kim D, Paggi JM, Park C, Bennett C, Salzberg SL (2019). Graph-based genome alignment and genotyping with HISAT2 and HISAT-genotype. Nat. Biotechnol..

[68] Li H (2009). The sequence Alignment/Map format and SAMtools. Bioinformatics.

[69] Anders S, Pyl PT, Huber W (2015). HTSeq–a Python framework to work with high-throughput sequencing data. Bioinformatics.

[70] Ramírez F (2016). deepTools2: a next generation web server for deep-sequencing data analysis. Nucleic Acids Res..

[71] Huang DW, Sherman BT, Lempicki RA (2009). Systematic and integrative analysis of large gene lists using DAVID bioinformatics resources. Nat. Protoc..

[72] Langmead B, Trapnell C, Pop M, Salzberg SL (2009). Ultrafast and memory-efficient alignment of short DNA sequences to the human genome. Genome Biol..

[73] McLean CY (2010). GREAT improves functional interpretation of *cis*-regulatory regions. Nat. Biotechnol..

[74] Heinz S (2010). Simple combinations of lineage-determining transcription factors prime *cis*-regulatory elements required for macrophage and B cell identities. Mol. Cell..

[75] Purcell S (2007). PLINK: a tool set for whole-genome association and population-based linkage analyses. Am. J. Hum. Genet..

[76] Wang K, Li M, Hakonarson H (2010). ANNOVAR: functional annotation of genetic variants from high-throughput sequencing data. Nucleic Acids Res..

[77] Jin LH (2024). figshare.

