# Supplementary material for: Genome-wide profiling of angiogenic cis-regulatory elements unravels cis-regulatory SNPs for vascular abnormality
Source: Sci Data. 2024 May 8;11:467. doi: 10.1038/s41597-024-03272-6 (PMC11078952; doi:10.1038/s41597-024-03272-6)
Supplement: Supplementary file 1 — Supplementary Information [file 41597_2024_3272_MOESM1_ESM.pdf]

# **Genome-wide profiling of angiogenic *cis*-regulatory elements unravels *cis*-regulatory SNPs for vascular abnormality**

Lihui Jin[†, *], Zhenyuan Han[†, *], Xiaotong Mao, Jieru Lu, Bingqian Yan,

Yiwen Lu, Lili Liang, Lin Wang*, Yu Yu*, Kun Sun*

**Supplementary Information**

**Supplementary Figures 1-2**

**Supplementary Table 1**

a

| H-0 | H-1 | H-4 | H-12 |
|---|---|---|---|
| AQP1 | BMP2 | CDH11 | CD34 |
| BMP4 | CCL2 | CDK6 | EIF2B3 |
| BMP6 | ICAM1 | ETV5 | HDAC5 |
| EDN1 | IL1A | HMGA2 | HDAC9 |
| ICAM2 | JAG1 | MEF2A | NR5A2 |
| IL33 | PROX1 | MEF2C | PDGFA |
| NRG1 | SOX7 | WWTR1 | PDGFD |
| ZEB2 | TSHZ1 | ZEB1 | PRDM1 |

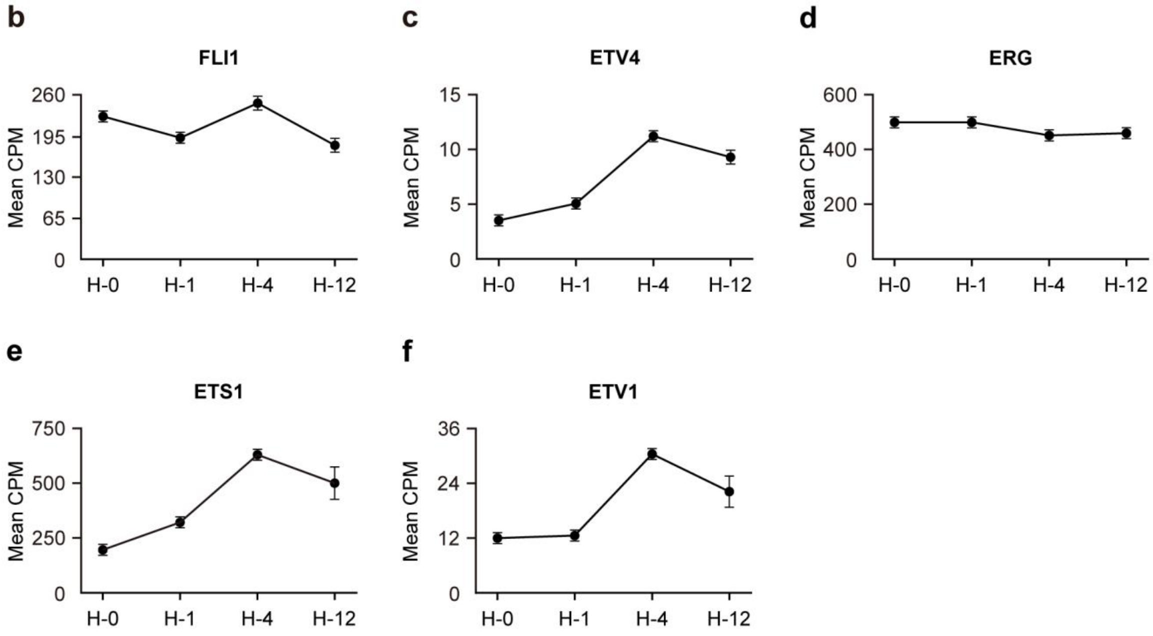


**Supplementary Fig.1** RNA-seq reanalysis. (**a**) Typical DEGs specifically enriched in H-0, H-1, H-4 and H-12. (**b-f**) Normalized expression levels of *FLI1* (**b**), *ETV4* (**c**), *ERG* (**d**), *ETS1* (**e**) and *ETV1* (**f**) during VEGFA stimulation, respectively. Data represented means ± SEM (n = 2 per group).

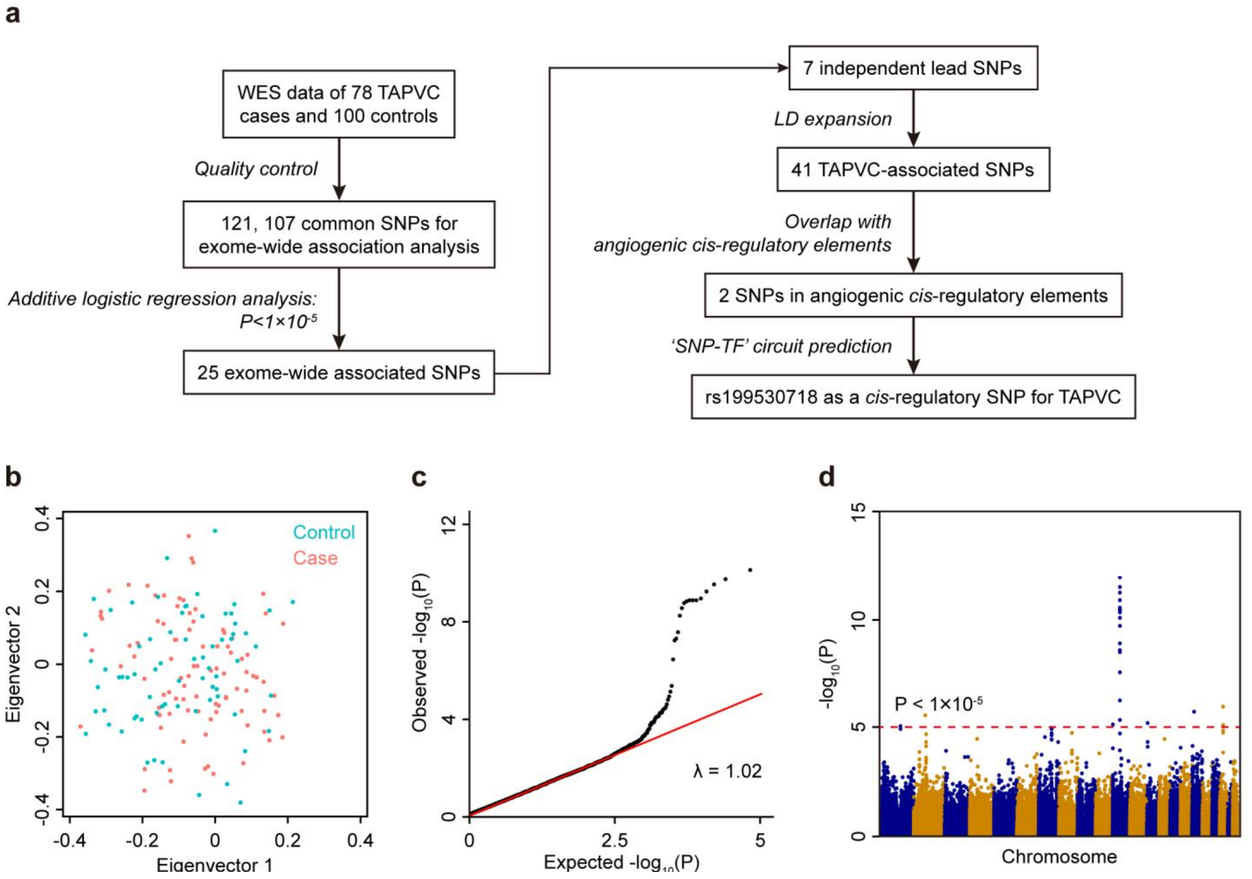


**Supplementary Fig.2** Exome-wide association analysis. (**a**) Flow chart showing the concise pipeline used to identify *cis*-regulatory SNPs for TAPVC. (**b**) PCA plot showing the first two eigenvectors of 78 TAPVC cases and 100 controls. (**c**) Q-Q plot of the SNP-based associations with TAPVC. (**d**) Manhattan plot showing the $-\log_{10}$-transformed *p* value of each SNP on the *y* axis and base-pair positions along autosomal chromosomes on the *x* axis. The red dashed line indicates exome-wide association ($p < 1\times10^{-5}$).

**Supplementary Table 1** Accession of public datasets used in the study.

| Sequencing type | Samples | Reference | Accession |
|---|---|---|---|
| WES | TAPVC cases | Shi et al. | / |
| | Healthy controls | | |
| DNase-seq | H-0 | Zhang et al. | GSE41166 |
| | H-1 | | |
| | H-4 | | |
| | H-12 | | |
| RNA-seq | H-0 | Zhang et al. | GSE41166 |
| | H-1 | | |
| | H-4 | | |
| | H-12 | | |
| ChIP-seq | H3K27ac | Wang et al. | GSE109626 |
| | H3K4me3 | | |
